# Unraveling the Mechanism and Practical Implications of the Sol-Gel Synthesis of Spinel LiMn_2_O_4_ as a Cathode Material for Li-Ion Batteries: Critical Effects of Cation Distribution at the Matrix Level

**DOI:** 10.3390/molecules28083489

**Published:** 2023-04-15

**Authors:** Oyunbayar Nyamaa, Gyeong-Ho Kang, Sun-Chul Huh, Jeong-Hyeon Yang, Tae-Hyun Nam, Jung-Pil Noh

**Affiliations:** 1 Department of Smart Energy and Mechanical Engineering, Gyeongsang National University, Tongyeong-Haeanro 2, Tongyeong 53064, Republic of Korea; 2 Department of Materials Engineering and Convergence Technology, Gyeongsang National University, Jinju-daero 501, Jinju 52828, Republic of Korea; 3 Department of Mechanical System Engineering, Gyeongsang National University, Tongyeong-Haeanro 2, Tongyeong 53064, Republic of Korea

**Keywords:** spinel LiMn_2_O_4_, sol-gel synthesis mechanism, chemical homogeneity, chelated complex, cross-linked gel, Li-ion battery

## Abstract

Spinel LiMn_2_O_4_ (LMO) is a state-of-the-art cathode material for Li-ion batteries. However, the operating voltage and battery life of spinel LMO needs to be improved for application in various modern technologies. Modifying the composition of the spinel LMO material alters its electronic structure, thereby increasing its operating voltage. Additionally, modifying the microstructure of the spinel LMO by controlling the size and distribution of the particles can improve its electrochemical properties. In this study, we elucidate the sol-gel synthesis mechanisms of two common types of sol-gels (modified and unmodified metal complexes)—chelate gel and organic polymeric gel—and investigate their structural and morphological properties and electrochemical performances. This study highlights that uniform distribution of cations during sol-gel formation is important for the growth of LMO crystals. Furthermore, a homogeneous multicomponent sol-gel, necessary to ensure that no conflicting morphologies and structures would degrade the electrochemical performances, can be obtained when the sol-gel has a polymer-like structure and uniformly bound ions; this can be achieved by using additional multifunctional reagents, namely cross-linkers.

## 1. Introduction

Li-ion batteries (LIBs) are used in a variety of applications, because they offer several benefits, including high energy density, low weight, low self-discharge, high cycle life, and environmental friendliness, over other types of batteries [1]. LIBs can be employed in frequently used products with longer life expectations. However, their battery performance needs to be improved further to meet the requirements of developments in modern technology. During the battery development process, the main performance parameters such as voltage, capacity, cycle life, self-discharge, temperature range, safety, and cost are considered individually. Therefore, developing a high-performance battery or improving the abovementioned performance parameters requires the development of high-performance individual components of the battery. Among the battery components, the cathode is the main component that affects battery capacity. Lithium cobalt oxide (LCO) is the most commonly used cathode material for LIBs owing to its high energy density and high operating voltage, making it a popular choice for use in portable electronic devices such as smartphones and laptops. However, LCO has certain drawbacks including high cost, low cycle life, and a tendency to overheat if it is rapidly charged or discharged [2]. To address these problems, other cathode materials, such as lithium nickel manganese cobalt oxide (NMC) [3], lithium manganese oxide (LMO) [4], and lithium iron phosphate (LFP) [4,5] have been developed. These materials have different trade-offs between energy density, cycle life, and safety and are used in different types of applications based on their specific properties. Among them, the unique 3D-tunnel-structured spinel LMO has been considered to be the most promising alternative cathode material for the new generation of lithium-ion batteries owing to its high capacity, good rate performance, non-toxicity, and ease of manufacturing [6]. In addition, LMO is relatively inexpensive and abundant, making it attractive for use in many applications. However, the operating voltage and battery life of spinel LMO need to be improved to expand its applications [7]. One way to improve the low operating voltage of spinel LMO is to modify the composition of the material. This can be achieved by replacing some of the manganese in the spinel with another element that has higher operating voltage levels; for example, cobalt or nickel [4,7]. Another method to improve the voltage of spinel LMO is to add dopants, which are small amounts of other elements, to the material [8]; this can alter the electronic structure of spinel LMO and increase its operating voltage. In addition, modifying the microstructure of spinel LMO by controlling the size or distribution of the particles can also improve its electrochemical properties [9,10,11,12,13]. Several factors can reduce the cycle life of LMO cathodes: LMO cathodes can be sensitive to high operating voltages, temperatures, and use poor quality materials or manufacturing processes [14,15,16]. Therefore, the first priority is to produce LMO of high purity with uniform particle and size distribution to improve the structure and morphology of the LMO cathode, owing to which the electrochemical performance can be improved [17,18]. For example, smaller particles have a larger surface-area-to-volume ratio, which can improve the kinetics of charge and discharge reactions (Li-ion diffusion), resulting in improved electrochemical performance [8,19,20]. In addition, homogeneous particles with a narrow size distribution can reduce the particle-to-particle variability and improve the consistency of the electrochemical performance. However, homogeneous particles with a high degree of agglomeration can reduce the effective surface area of the particles, leading to reduced electrochemical performance [21,22]. Similarly, particles with defects or impurities can lead to electrochemical degradation, resulting in a reduced performance and cycle life. In the synthesis of high-purity particles using wet chemical methods, the choice and quality of solvents, reagents, and reaction conditions are critical for achieving high purity. Controlling the purity of the starting materials, as well as minimizing the presence of impurities during the reaction and purification steps, is essential for achieving high-purity particles. In addition, it is crucial to carefully control the reaction conditions (such as temperature, pressure, and pH) to prevent unwanted side reactions, and to clean the product thoroughly to remove residual impurities. In addition, for sol-gel synthesis, it is important to mix the starting materials at a stoichiometric ratio to conduct a residual-free reaction [15,23,24,25]; therefore, the synthesis of active materials is very important. There are several methods for synthesizing spinel LMO. One common method is solid-state synthesis, in which the starting materials are mixed and heated to high temperatures in a furnace to form the desired product. Another method is sol-gel synthesis, in which the starting materials are dissolved in a solvent to form a gel, which is then heated to remove the solvent and form a spinel. Other methods for synthesizing spinel LMO include hydrothermal, coprecipitation, and solvothermal synthesis [8,26]. Among these, the sol-gel method is well-suited to meet the above requirements.

Some advantages of sol-gel synthesis include a low calcination temperature, short processing time, and the formation of submicron-sized particles with a narrow particle-size distribution [27]. In addition, it does not produce harmful by-products or require high temperatures, making it a safer alternative to other synthesis methods [27]. The sol-gel synthesis of materials is a multistep process that involves the conversion of a precursor solution into a sol or gel, and the subsequent conversion of the sol-gel to an oxide compound [28,29]. Citric acid-assisted sol-gel synthesis, which uses citric acid as a chelating agent, is the most commonly used method for LMO production. Furthermore, it has been reported that the use of ethylenediaminetetraacetic acid (EDTA), an amino polycarboxylic acid, as a chelating agent improves the electrochemical properties by forming metal complexes with different structures of complex precursors and providing better conditions for the formation of LMO [10]. Moreover, many studies have reported that by manipulating the sol-gel structure using different chelating reagents, the shape and size distribution of LMO particles can be controlled during LMO formation [28,30,31]. Chemical homogeneity with respect to the distribution of cations in the sol-gel often determines the compositional homogeneity of the final powder. Therefore, it is crucial to design an appropriate precursor solution for the formation of a homogeneous multicomponent gel without the occurrence of phase segregation during sol-gel formation [29]. One strategy to overcome this problem and obtain a homogeneous multicomponent gel is to obtain a sol-gel with an essentially polymer-like structure and to uniformly bind the ions using multifunctional reagents such as cross-linkers [32,33]. The influence of crosslinking agents or organic polymer gels changes the structure of the sol-gel at the molecular level, which reduces the segregation of particular metal ions via the combustion of polymer intermediates and ensures the compositional homogeneity of LMO powders [29]. However, although LMOs prepared using these methods have been extensively studied, the critical effects of cation distribution at the matrix level are yet to be reported.

In this study, we described the synthesis mechanism of two commonly used types of sol-gels: chelate and organic polymeric gels [34]. Their structural, morphological, and electrochemical properties when prepared under the same reaction conditions were investigated, and the theoretical and practical implications of the results were discussed. It is hoped that further development of the sol-gel synthesis process may facilitate methods such as coating, element substitution, and fusion for solving challenges pertaining to the use of LMO as a cathode material. Furthermore, the ordered distribution of cations in the sol-gel phase was achieved using key reagents, such as highly structured additional reagents of multifunctional or chelating reagents and does not rule out finding LMO in the sol-gel phase as well.

## 2. Results

### 2.1. Structure and Morphology

**Figure 1:**
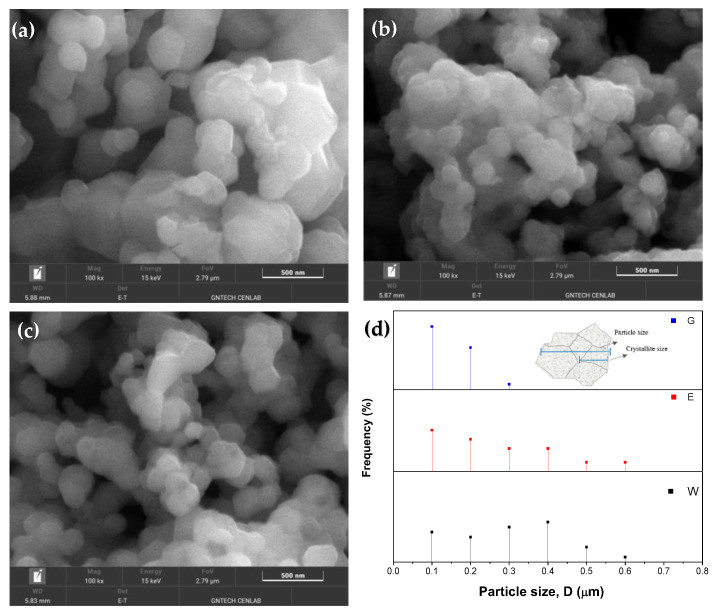
SEM images of (**a**) LMO-W, (**b**) LMO-E, and (**c**) LMO-G powders. (**d**) Particle-size distribution for all samples.

**Figure 2:**
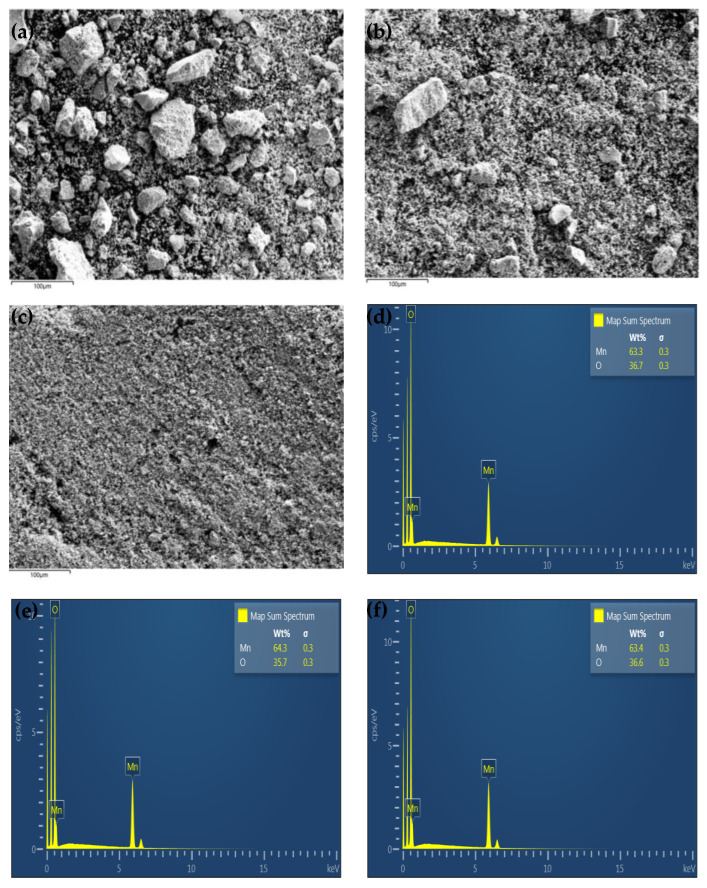
EDS electron images of (**a**) LMO-W, (**b**) LMO-E, and (**c**) LMO-G. EDS spectra of (**d**) LMO-W, (**e**) LMO-E, and (**f**) LMO-G samples.

Figure 1 shows the SEM images of the LMO-W, LMO-E, and LMO-G powders. All three powders exhibited regular octahedral morphologies and high crystallinity and consisted of a few agglomerates with a large size distribution. While the LMO-W and LMO-E powders exhibited close dense agglomeration, the LMO-G sample exhibited uniform and weak agglomeration owing to the uniform distribution of cations in the cross-linked structured polymer gel. Although the grain growth process is mainly dependent on the annealing conditions, it has been reported that, under the same conditions, the crystal growth process changes with the distribution of cations in the matrix [35]. The average size of the crystalline grains was estimated to be 351 nm for LMO-W and 312 nm for LMO-E but was drastically lower, 219 nm, for the LMO-G powder. As can be seen from the particle size distribution in Figure 1d, which was determined using the ImageJ software [36], the LMO-W sample had a measured particle size range of 189 nm and 611 nm, with more than 66% of the distribution being between ~200 nm and ~400 nm because of agglomeration; meanwhile the particle sizes in the LMO-E sample varied between 176 nm and 598 nm, with ~70% of the particles being between 100 nm and 300 nm. In the LMO-G sample, the particle sizes ranged between 133 nm and 365 nm with 86% of the particles being distributed between approximately 100 and 200 particles, that is, the particles were almost monodispersed which is a narrower and, therefore, improved particle size distribution compared with the LMO-W and LMO-E samples. In addition, the dispersion states of the powders were observed from the depth differences in the electron images (Figure 2a–c). For LMO-W and LMO-E, the grains aggregated, causing the appearance of large-size particles in some parts, while for sample LMO-G, no apparent difference in particle size could be observed. The compositions of the powders were analyzed using EDS, as shown in Figure 2d–f; all three samples were composed of O and Mn at an atomic weight ratio of approximately 2:1, indicating a stoichiometric composition of LMO. Table A1 presents a comparison of the samples obtained using the sol-gel method under similar conditions, except for the addition of a small amount of ethanol or ethylene glycol. The synthesis was performed without any additional catalytic reagents or processing, using only pure citric acid and ethanol or ethylene glycol as monomers for polymerization in aqueous media. 

**Figure 3:**
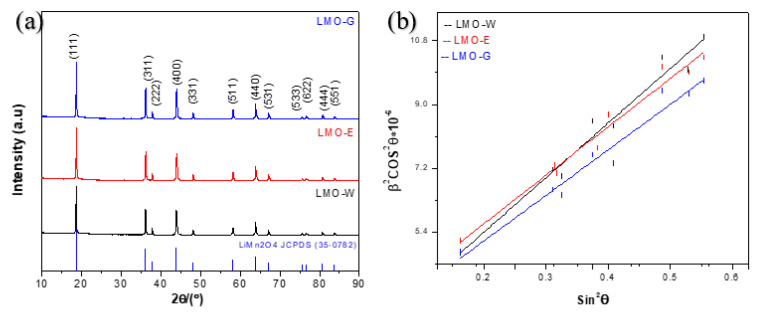
XRD patterns of (**a**) LMO samples. (**b**) Analysis of the full width at half maximum β of the Bragg peaks.

To determine the effect of the chemical environment on crystallinity, XRD studies were performed on three types of powders prepared under different chemical modification conditions, that is, by varying the monomer of polymerization. Figure 3 shows the XRD patterns of the LMOs and the full width at half maximum β of the Bragg peaks. The corresponding lattice constants, a, are summarized in Table 1, and are compared to that of the cubic spinel LMO obtained from the JCPDS card. A similar XRD pattern was obtained for all samples, and their diffraction peaks could be indexed to a single cubic LMO Fd-3m space group. In addition, only corresponding diffraction peaks were observed, indicating that the samples were highly pure without undesirable impurities. If the sol-gel matrix is not properly removed during calcination, it can leave behind carbonaceous residues that could interfere with the formation of the spinel phase or affect its properties [24]; therefore, carbonaceous residues were completely removed by calcinating the samples at 800 °C, as reported in the literature [24], which was confirmed by the XRD results. The intensity ratios I_(111)_/I_(311)_, I_(311)_/I_(400)_, I_(400)_/I_(111)_, and I_(440)_/I_(111)_ from the crystal facet XRD patterns of spinel LMO powder provide information about the crystal structure and orientation of the material [37]; the I_(111)_/I_(311)_ ratio indicates the growth of the (111) crystal plane, whereas the (440) and (400) crystal facets correspond to the (110) and (100) crystal planes, respectively [37]. The intensity ratios were calculated (Table 1) for all samples to compare the crystal structure plane orientations. The I_(111)_/I_(311)_ ratios showed similar values, with values of 1.88 for LMO-W and 1.81 for LMO-E and LMO-G; however, the value for the LMO-W sample being slightly higher indicates that the powder had a preferential orientation along the (111) crystallographic direction [37,38,39,40]. The (111) crystal planes of the spinel LMO particles being predominantly oriented in a particular direction can lead to anisotropic properties, such as different electrochemical performances in different crystallographic directions. In contrast, comparing the I_(400)_/I_(111)_ intensity ratios, the value for the LMO-W sample was slightly lower than those for the LMO-E and LMO-G samples, indicating that the (100) crystal plane was dominant, which provides more favorable support for Li^+^ transport kinetics [41]. However, comparing the I_(440)_/I_(111)_ intensity ratios, the (110) crystal plane was dominant for LMO-E, while LMO-W and LMO-G had the same values. The I_(311)_/I_(400)_ ratios of the samples were in the range of 1.02–1.09, which indicates that the samples were similar to each other, and that the [Mn_2_]O_4_ spinel framework is stable [42,43]. These small differences in crystallinity and crystal structure indicate that the (100) crystal plane, which is favorable for Li^+^ transport kinetics, is predominant in the LMO-E and LMO-G samples, which contain carbon atoms in the sol-gel matrix; this may be because of the small increase in temperature caused by the production of CO_2_ and CO during combustion [41]. Furthermore, the lattice strain can provide information on the crystal structure of a material. Lattice strain can be evidenced by the use of the Scherrer formula [10].

β^2^cos^2^(θ) = 16e^2^sin^2^(θ) + (K^2^λ^2^/L^2^), (1)

where β is the full width at half maximum, θ is the diffraction, e^2^ is a local strain (defined as Δd/d, where d is the interplanar spacing), L is the crystallite size, and K = 0.88 for a spherical crystallite shape. The plot of β^2^cos^2^ (θ) as a function of sin^2^ θ, the slope e^2^, the intercept K^2^λ^2^/L^2^, and the coherence length L were used to calculate the strain e^2^. As presented in Table 1, the lattice strain was calculated to be 3.57 × 10^–6^ for the LMO-W sample, 2.89 × 10^–6^ for the LMO-E sample, and the lowest value of 2.62 × 10^–6^ for the LMO-G sample. From the local strain result, it appears that there is more stress on a larger scale, but it also verified that the difference in strain arose from differences in the internal electronic structures and crystallinity of the synthesized samples. For this study, all the changes in structures between samples can be explained by the synthesis process, and, further, by considering the mechanism of the citric acid-assisted sol-gel synthesis from the initial sol-gel formation stage to the final LMO extraction stage. 

### 2.2. Mechanism of Sol-Gel Synthesis

**Figure 4:**
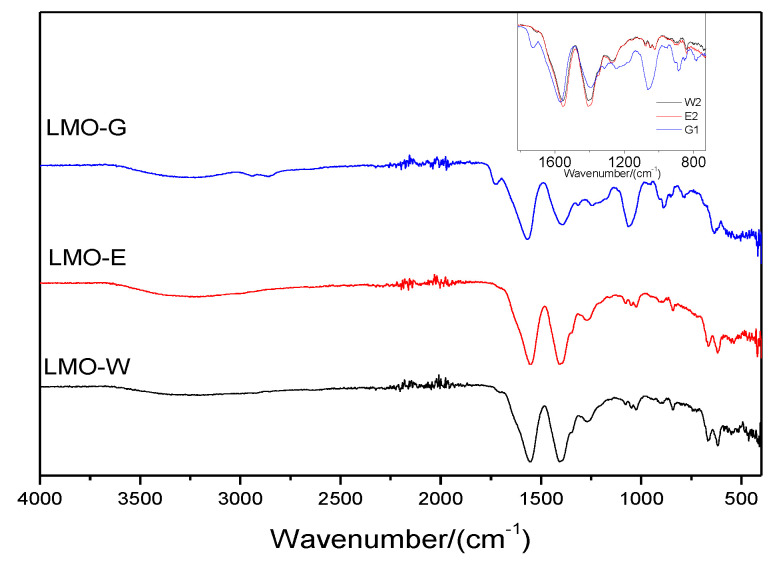
FTIR spectra of obtained LMO sol-gels.

To determine the functional groups and crystallinity of the gel during the sol-gel formation stage for the synthesis mechanism, FTIR analysis was performed on the sol-gel form of each sample; hereinafter, the sol-gel prepared from LMO-G will be referred to as G-G, from LMO-W as G-W, and from LMO-E as G-E. Figure 4 shows the FTIR spectra of the sol-gels obtained. All three sol-gels exhibited a broad absorption band between 3200 cm^−1^ and 3400 cm^−1^, indicating the presence of carboxyl and water-derived hydroxyl groups [44]. The characteristic peaks at 2856 cm^−1^ (G-G) and 1030 cm^−1^ to 1075 cm^−1^ (G-W, G-E, and G-G) are attributed to the bending vibrations of the C–O, C–C, and C–O–C groups [45]. In addition, two strong bands were observed from 1552 cm^−1^ to 1408 cm^−1^ for G-W, 1553 cm^−1^ to 1408 cm^−1^ for G-E, and 1568 cm^−1^ to 1393 cm^−1^ for G-G. These indicate the symmetric and asymmetric stretching vibrations of the C=O bond in metal carboxylates, thereby confirming the chelation of metal ions by citric acid [31,44,46]. For the G-G gel, a characteristic peak was observed at 1725 cm^−1^, which was not observed for G-W or G-E; this indicated the formation of the cross-linked polymer, and it is obvious that ethylene glycol was polymerized by cross-linking the ester through two carboxyl groups [47]. In comparison, it can be concluded that the ${\nu_{a(COO)}}^{-}$ and ${\nu_{\left(COO\right)}}^{-}$ of free carboxy anions are mixed with carboxy groups of the unionized form in the G-W sol-gel and G-E. For G-G, the presence of extended peaks attributed to the symmetric and asymmetric stretching vibration of C=O indicated a decrease in the carboxyl groups of the unionized form [46,48].

Based on the literature and FTIR results, the synthesis mechanism of the citric-assisted sol-gel method can be explained by the reaction between cations, citric acid, solvent, and a monomer of sol-gel polymerization, as follows. Mechanistic pathways were considered for each sample tour. For LMO-W, the reactions observed when lithium acetate, manganese acetate, and citric acid are dissolved in water and then mixed can be expressed using Equations (2)–(4). In Equation (2), when manganese acetate is dissolved in water, the acetate ions [CH_3_COO^−^] separate from the manganese ions [Mn^2+^] and react with water molecules to form acetic acid and hydroxide ions [OH^−^]. The hydroxide ions then react with the manganese ions to form manganese hydroxide. However, this reaction depends on the pH of the solution, and can then be controlled by changing the pH [24]. In Equation (3), when lithium acetate is dissolved in water, the acetate ions [CH_3_COO^−^] separate from the lithium ions [Li^+^] and react with water molecules to form acetic acid and hydroxide ions [OH^−^]. Citric acid is a weak organic acid that dissolves in water to produce hydrogen ions [H^+^] and citrate ions (Equation (4)) [28,34,46].

Mn(CH_2_COOH)_2_ + 2H_2_O = Mn^2+^ + 2OH^−^ + 2CH_2_COO^−^ + 4H^+^, (2)

LiCH_2_COOH + H_2_O = Li^+^ + OH^−^ + CH_2_COO^−^ + 2H^+^, (3)

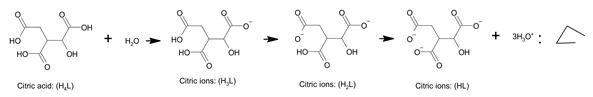
(4)

Generally, metal ions can form coordination complexes with organic compounds such as citric acid. Therefore, [Li^+^] forms lithium citrate, whereas [Mn^2+^] forms an independent chelate compound. According to the literature, the chief chelate compound of manganese citrate is lgβ = 8 (MnH_2_L), which means that the chelate compound of manganese citrate is mostly a chelate compound of a single molecule [46,49,50]. The reaction equations for the formation of the corresponding lithium citrate and manganese chelate complexes can be expressed using Equations (5) and (6).

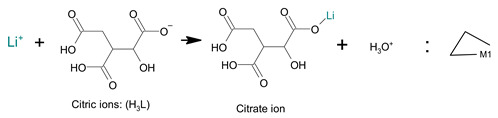
(5)

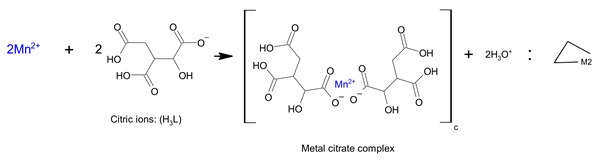
(6)


Therefore, in the case of sample A, the sol-gel structure was a complex of metal ions, and metal ions were dispersed through this complex (Equation (7)).

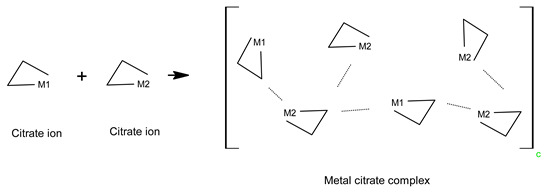
(7)


In the case of B, the dissolution process of sample B was the same as that of sample A, so the reaction mechanism was the same. In the next step, the esterification reaction between the metal citrate complex and ethanol occurred through a series of reactions with tri-ethyl citrate, as expressed in Equations (8) and (9) [24,51]. The ester is not a polymer, but a large molecule made up of repeating units or monomers. Therefore, the sol-gel structure of sample B was a non-polymerizable metal complex [28], which permits less compositional homogeneity [52]. The general form of the sol-gel structure can be expressed using Equation (10).

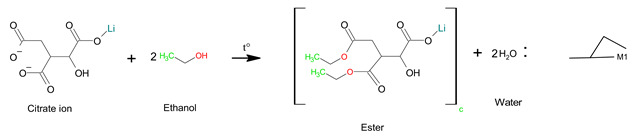
(8)

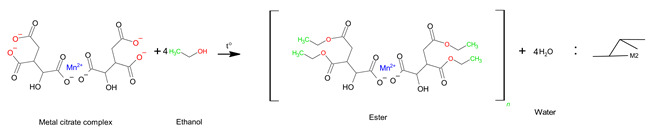
(9)

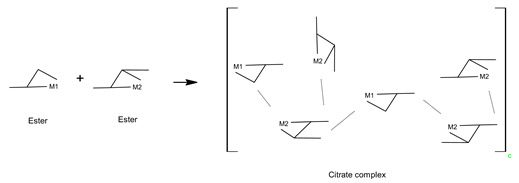
(10)


In the case of sample C, the metal ions and citric acid were dissolved in water; the reaction was the same as that for sample A. After that ethylene glycol was mixed with the resulting metal citric complex solution, and the esterification reaction proceeded, as shown in Equation (11). Because polymerization occurs at this stage while emitting 120 °C heat, the choice of a monomeric compound with two more alcohol groups (–OH) indicates that it is possible to obtain a cross-linked polymer gel (Equation (12)). Therefore, sol-gels with three-dimensional network structures with a high dispersion of metal cations over short distances can be achieved using polymers containing two (–OH) groups [52]. This method of polymerization, which involves the formation of a polymer gel using citric acid and ethylene glycol as the monomers, is known as the Pechini method [28,46,52].

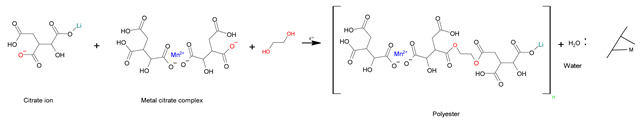
(11)

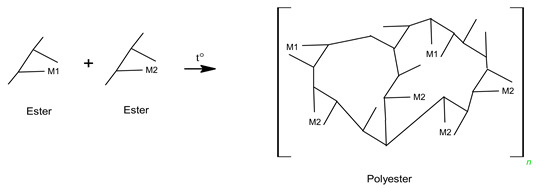
(12)


The process of gelation is completed when polymerization is completed through condensation, following which the next process of LMO formation begins by combusting the distributed cations on the atomic scale in the organic matrix. The combustion process takes place in two stages at 450 °C and 800 °C; at temperatures below 420 °C, dehydration and decarboxylation (removal of the organic) occur. The cations are uncoordinated from their ligands at this stage, and the oxides and spinel structures are mixed in the system; the related equations are shown in Equations (13)–(15). Then, during thermal decomposition at 800 °C, stable oxides are formed, further organic ligand residues are completely removed, and the previously formed spinel LMO grows, resulting in a pure LMO structure (Equation (16)) [53].

(13) $$2\mathrm{Li}(C_{5+n}H_{n}O_{4})+\frac{\left(13+5n\right)}{2}O_{2}\;\to \;{\mathrm{Li}}_{2}O+(10+2n){\mathrm{CO}}_{2}+nH_{2}O,$$

(14) $$2\mathrm{Mn}(C_{5+n}H_{n}O_{4})+\frac{\left(15+5n\right)}{2}O_{2}\;\to \;{\mathrm{Mn}}_{2}O_{3}+(10+2n){\mathrm{CO}}_{2}+nH_{2}O,$$

(15) $$\mathrm{Mn}(C_{5+n}H_{n}O_{4})+\frac{20+5n}{4}O_{2}\;\to \;{\mathrm{MnO}}_{2}+(5+n){\mathrm{CO}}_{2}+\frac{n}{2}H_{2}O,$$

Li_2_O + Mn_2_O_3_ + 2MnO_2_ → 2LiMn_2_O_4_, (16)

### 2.3. X-ray Photoelectron Spectroscopy (XPS) Analysis

**Figure 5:**
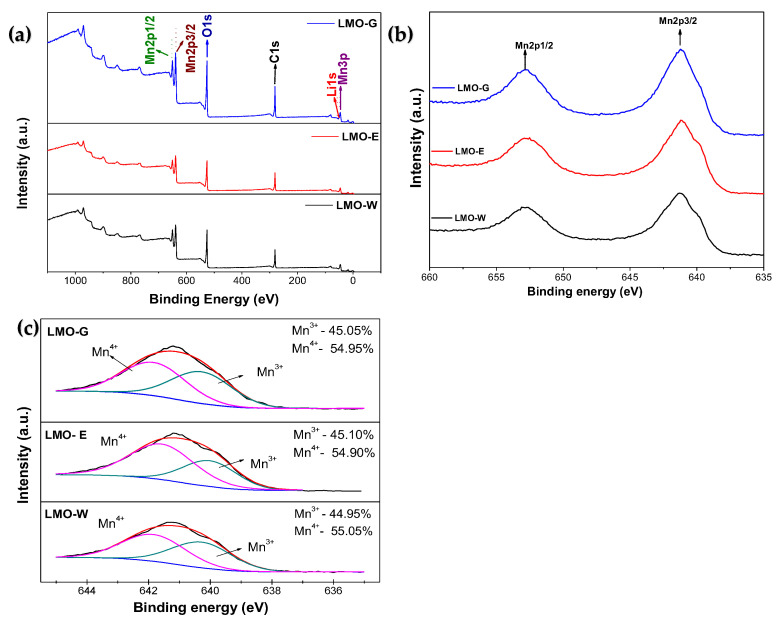
XPS spectra for LMO samples: (**a**) full spectra; (**b**) Mn 2p peaks of LMO samples; (**c**) deconvoluted profiles of Mn 2p_3/2_ XPS spectra.

A metal chelate complex containing a high number of carbon atoms cannot reduce the Mn content in spinel LMO during calcination; however, it can control the Mn oxidation state and improve the electrochemical performance of the material by increasing the temperature [24]. As shown in Equations (4), (7) and (8), it depends on the content of carbon atoms contained in the sol-gel structure, and the G-G and G-E structures contain more carbon atoms than the G-W structure because of the additional of ethanol and ethylene glycol contents. To confirm this, XPS analysis was performed for each sample, as XPS can provide information about the oxidation state of a particular element in a sample using semiquantitative estimation. The Mn 2p region is typically used to analyze the oxidation state of Mn because it is more sensitive to the valence state of the element than the Mn 3p region. The Mn 2p region has two peaks, one associated with the Mn 2p_3/2_ state and the other with the Mn 2p_1/2_ state. The energy difference between the two peaks can be used to determine the oxidation state of Mn: the Mn 2p_3/2_ peak generally shifts to higher binding energies as the oxidation state of Mn increases, owing to which the position of the Mn 2p_3/2_ peak can be used to distinguish between [Mn^3+^] and [Mn^4+^]. Figure 5 shows the XPS spectra of the LMOs samples. The energy band at approximately 51 eV was assigned to Mn 3p and Li 1s, the band at approximately 530 eV to O 1s, and the bands at approximately 641 eV and 653 eV to Mn 2p_3/2_ and Mn 2p_1/2_, respectively (Figure 5a,b). Comparing the Mn 2p_1/2_ and Mn 2p_3/2_ peaks in the XPS spectra of the samples (Figure 5b), the peaks for LMO-E (641.21 eV and 652.91 eV) and LMO-G (641.20 eV and 652.71 eV) were at lower binding energies compared with those for LMO-W (641.31 eV and 653.11 eV). If the peak associated with the Mn 2p_3/2_ state shifts to a lower binding energy, it suggests that the electron density around the Mn nucleus has increased, which is consistent with a decrease in the oxidation state [37,54]. From the deconvoluted results in Figure 5c, the contents of [Mn^3+^] and [Mn^4+^] are similar for all samples: 55.05% and 44.95% for LMO-W, 54.90% and 45.10% for LMO-E, 54.95% and 45.05% for LMO-G, respectively. Therefore, from the deconvoluted results, it can be concluded that the total amount of carbon used in the synthesis was insufficient to change the oxidation state of the Mn ions. Moreover, the [Mn^4+^] and [Mn^3+^] content ratio ([Mn^4+^]/[Mn^3+^]) of the samples is similar at 1.22, 1.23, and 1.23 for LMO-W, LMO-E, and LMO-G, respectively, which indicates that the J–T distortion was uniform in all samples [37]. 

### 2.4. Electrochemical Characterization

**Figure 6:**
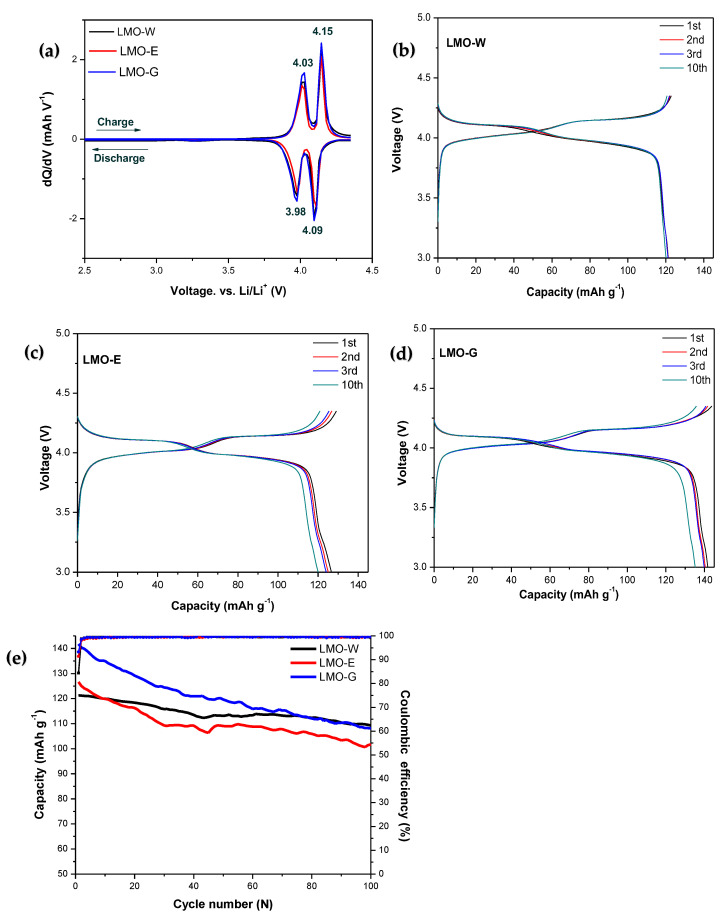
(**a**) dQ/dV plots of LMO-W, LMO-E, and LMO-G powders. Charge/discharge capacity profiles of (**b**) LMO-W, (**c**) LMO-E, and (**d**) LMO-G. (**e**) Discharge cycling performance of LMOs after 100 cycles over a cut-off voltage range of 3.0 V to 4.35 V at 0.5 C.

The electrochemical intercalation/deintercalation behaviors of the spinel LMO samples were examined through differential capacity curves (dQ/dV) and are shown in Figure 6a. In the dQ/dV curves for all the samples, two pairs of separated redox peaks can be clearly observed at 4.01 V and 4.15 V, in describing the way that Li-ions are extracted from 8a tetrahedral sites and inserted into the spinel phase in a two-step transition [11,12,36,55]: the first step can be expressed as LiMn_2_O_4_ → Li_(1−x)_Mn_2_O_4_ + xLi^+^ + 0.5e^−^ (x < 0.5), and the second as Li_(1−x)_Mn_2_O_4_ → 2MnO_2_ + (1 − x)Li^+^ + (1 − x)0.5e^−^ (x > 0.5) is a fully delithiated system (MnO_2_) [56,57,58]. The highly symmetrical redox peaks indicate high reversibility in the two-step oxidation or reduction process [9] and the results for all synthesized samples are in good agreement with those reported in the literature. In comparison, the separation of redox peak potentials (∆ϕ_p_) for all samples showed similar characteristics, and the peak intensity of LMO-G was slightly higher than that of the other samples, indicating higher electrochemical activity [56]. Figure 6 shows the charge/discharge profiles of LMO-W, LMO-E, and LMO-G between 3.0 V and 4.35 V at a current density of 0.5 C. The profiles for all three samples exhibit two similar consecutive plateaus at approximately 4.05/4.11 V and 4.19/4.15 V (Figure 6b–d). The plateaus are attributed to the two-step cubic phase transformations of LiMn_2_O_4_/Li_0.5_Mn_2_O_4_ and Li_0.5_Mn_2_O_4_/λ-MnO_2_ during oxidation and reduction, which is in good agreement with the differential capacity curves [59]. Figure 6 shows that the charge/discharge profiles of all three samples are in good agreement with the reported LMOs [10,60,61]. The samples differed in reversible initial charge/discharge: LMO-W, LMO-E, and LMO-G exhibited values of 122.8/121.3 mAh/g, 129.1/126.6 mAh/g, and the relatively high 143.7/141.6 mAh/g, respectively, along with corresponding Coulombic efficiencies of 98.78%, 98.05%, and 98.58%, respectively. For all samples, the initial Coulombic efficiency initially decreased owing to material dissolution and side reactions [51,62,63], but increased directly from the second cycle, reaching values of 99.21%, 98.68%, and 99.11%, respectively, indicating that the cycling stabilized. The LMO-W sample exhibited a relatively high initial Coulombic efficiency compared to LMO-E and LMO-G because of its larger particle size and lower specific surface area, which resulted in less Mn dissolution and a stable cycling performance, while the LMO-E and LMO-G samples exhibited larger electrode polarization [39]. Figure 6e shows the cyclic performance for all the samples at 0.5 C for 100 cycles. All three samples experienced severe capacity reduction owing to the well-known phenomenon of J–T distortion in spinel LMOs [51]. In terms of cycle performance, LMO-W was the most stable, with a capacity retention of 82% after the 100th cycle, whereas LMO-E and LMO-G experienced more noticeable capacity loss, with capacity retentions of 79% and 70%, respectively, at the 100th cycle [51,64]. Small particles can improve the electrochemical performance by enhancing the Li^+^ ion diffusion kinetics [41]; however, their high surface areas lead to a more unstable cycle life and capacity degradation after a limited number of cycles. This makes them more susceptible to phenomena such as grain growth, shrinkage, dissolution, and phase change owing to their high surface reactivity [65,66]. In addition, the Li^+^ ions are more tightly placed at the (100) and (110) planes than at the (111) planes, indicating that the exposed (100) and (110) planes on the surface are more favorable for facilitating Li^+^ transport kinetics [41]. Therefore, the higher capacities of LMO-G and LMO-E may also be supported by the dominance of the (100) crystal plane. 

**Figure 7:**
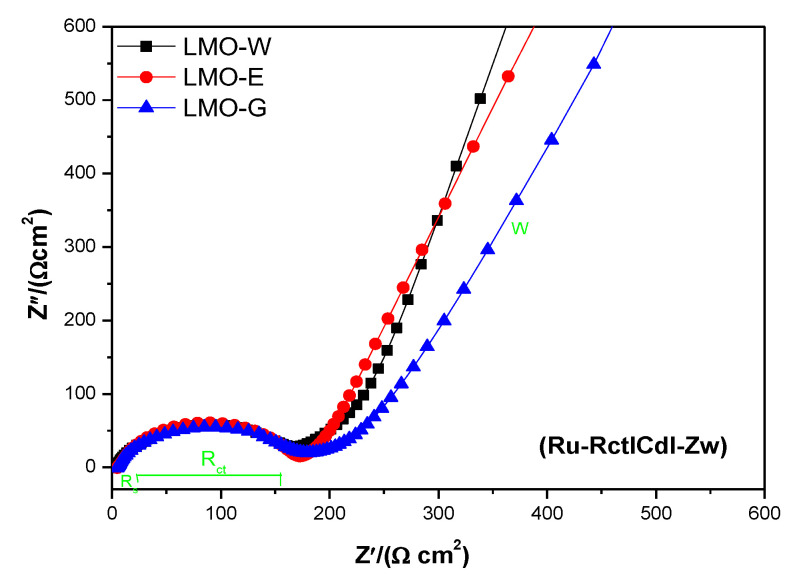
Electrochemical impedance spectra of the samples after 100 cycles of CC testing over a frequency range of 1 MHz to 10 MHz with a cut-off voltage of 3.0 V.

EIS analysis was performed to determine the electrochemical polarization and resistance of the cells and to harmonize it with the electrochemical performance of the samples. The Nyquist plots were investigated in the frequency range of 1 MHz to 10 MHz at 3.0 V after 100 cycles of the Galvanostatic charge–discharge tests (Figure 7). An equivalent circuit was used to analyze the EIS data. The semicircle is usually attributed to the combination of the solid/electrolyte interface film resistance and charge transfer impedance in the high-frequency region at the electrode surface, whereas an inclined line at low frequencies is described as a Warburg-type element reflecting the solid-state diffusion of Li into the bulk of the active materials [9,63,67]. The Nyquist plot shows a suppressed semicircle in the high-to-medium frequency region and an inclined line in the low frequency region. The curve shows that the solution resistance (R_s_) ranges from zero to the beginning of the semicircle and the charge transfer resistance (R_ct_) up to the end of the semicircle [63]. In terms of charge transfer resistance, LMO-W had the lowest resistance value of 167.8 Ω, which indicates the lowest electrochemical polarization, leading to a higher cycle performance [68]. It can be seen that the value for LMO-E (180.6) is larger than that for LMO-W. LMO-G exhibited the largest resistance value of 190.3 Ω, indicating the highest electrochemical polarization values, which resulted in a relatively lower cyclic performance [68]. The EIS results are in good agreement with the cycle performance results. Furthermore, owing to the higher surface area of the LMO-G sample compared to the LMO-W and LMO-E samples because of its smaller, more uniformly distributed particles, a higher number of defects occurred, such as the formation of a solid/electrolyte interface film, grain growth, shrinkage, dissolution, and phase change after cycling. This may be because the charge transfer resistance of LMO-G increased. Therefore, it can be seen here that the LMO-W and LMO-E samples have significantly fewer Li^+^ ion diffusion sites than the LMO-G sample. 

## 3. Discussion

The purity; shape; crystal size; crystal plane orientation; agglomeration of particles; presence of Li, Mn (Mn^3+^ and Mn^4+^), and O atoms; and the valence state of Mn ions are all critical characteristics that can influence the electrochemical performance of LMO cathode materials. Optimizing these characteristics can lead to improved battery performance, such as a higher capacity, better rate capability, and improved cycling stability. According to our results, using crosslinking agents in a polymer-like gel structure to react with the cations in the stoichiometric ratio has a positive effect on the structural properties of LMOs owing to uniform cation distribution at the matrix level. In the case of the samples synthesized using the sol-gel method under the same conditions, all samples exhibited similar LMO purity and contained Li, Mn (Mn^3+^ and Mn^4+^), and O atoms, but the uniformity and homogeneity of the particle size and crystal plane direction slightly differed. Based on the electrochemical results, it was found that modifying the sol-gel structure increased the surface area and improved the electrochemical properties of the sample by producing small homogenous LMO particles that were uniformly distributed throughout the highly structured gel structure (the difference in the sol-gel structures based on the mechanism of sol-gel synthesis is described in Section 2.2). However, from the XPS analysis, all samples suffered capacity loss during the cycles owing to J–T distortion by the dominant [Mn^3+^] ions. To reduce the J–T distortion effect, methods such as the doping strategy [69,70,71] or coating strategy [72,73] have been used, and our research also proves that the J–T distortion effect cannot be reduced by modifying the structure of the gel [51,74]. Therefore, the most successful effect of the modified sol-gel was its influence on the particle size and improvement of the homogeneity and uniformity of the LMO particles.

## 4. Materials and Methods

### 4.1. Materials

Spinel LMO active materials were prepared using the sol-gel method. Lithium acetate dihydrate (CH_3_COOLi 2H_2_O) and manganese acetate tetrahydrate ((CH_3_COO)_2_Mn 4H_2_O) (99.99%, Seohaean-ro Siheung-si, Gyeonggi-do Republic of Korea) were used as cations. Citric acid C_3_H_4_OH(COOH)_3_ (99.99%, Nihonbashihoncho, Chuoku, Tokyo Japan), ethylene glycol (HOCH_2_CH_2_OH) (99.99%, Seohaean-ro Siheung-si, Gyeonggi-do Republic of Korea), and ethanol (CH_3_CH_2_OH) (99.99%, South Korea) were used as chelating agents and polymerization monomers. Li foil and coin cells (CR 2032) were purchased from Wellcos Corporation, Ltd. (Toegye-dong, Chuncheon-si, Gangwon-do Republic of Korea) for assembling the battery. A solution of 1 M LiPF_6_ in ethylene carbonate (EC):diethyl carbonate (DEC) (1:1 vol. %), purchased from Soulbrain Co., Ltd. (Bundang-Gu, Seongnam, Gyeonggi-do Republic of Korea), was used as the electrolyte.

### 4.2. Synthesis of Spinel LMO Active Materials

**Figure 8:**
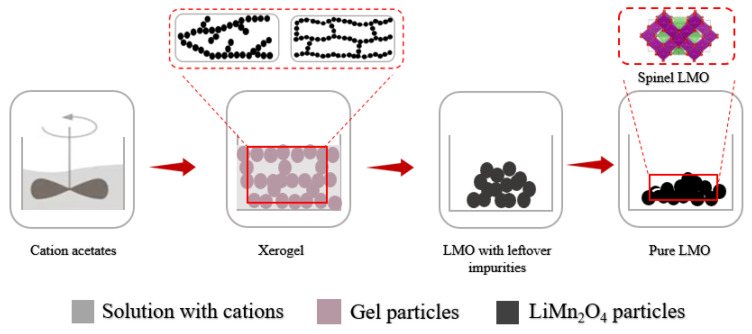
Schematic of the sample reparation process.

In this study, three types of samples were synthesized from the solutions acquired using water, ethanol, and ethylene glycol as the starting solutions, and the resulting powders were labeled as LMO-W, LMO-E, and LMO-G, respectively (Figure 8). To prepare LMO-W, lithium acetate and manganese acetate were dissolved in DI water just before saturation at a stoichiometric ratio of 1 mol and 2 mol, respectively. The solution was then added dropwise to a separately prepared 30% citric acid solution in water, such that the mol ratio of citric acid to total cations was 1:1. The mol ratio of total reagents is Li:Mn:citric acid = 1:2:3. The solution medium was buffered to approximately pH~4 with [OH^−^] ions using NH_4_OH, while the pH of the starting solution was approximately 3 owing to the citrate ions occurring mainly in presence of citric acid (pKa_1_ = 3.14) [24,75] as shown in the following reaction:

(17) $$K_{a}=\frac{{[C}_{6}H_{7}O_{7}{]}^{-}[{H]}^{+}}{{[C}_{6}H_{8}O_{7}]}$$

The second sample, LMO-E, was mixed with a solution prepared by dissolving cations in water (just before saturation) and then adding an aqueous solution of citric acid (30% concentration of solution) and ethanol, similar to the LMO-W sample. Cations and citric acid were used in the same amounts as in the LMO-W system, and 6 mol of ethanol was used based on the corresponding stoichiometric ratio. The mol ratio of total reagents is Li:Mn:citric acid:ethanol = 1:2:3:6. The prepared solution was initially buffered to pH 4. For LMO-G, the cations were dissolved in DI water and added to a citric acid solution dissolved in ethylene glycol at a mol ratio of 1:3. The mol ratio of total reagents is Li:Mn:citric acid:ethylene glycol = 1:2:3:9. The following steps of the sample sol-gel extraction process were performed under identical conditions. In the first step, lithium and manganese chelate compounds were formed in the solution. In the second step, the solution was further heated to 120 °C with stirring, and the esterification and polymerization processes were continued until a transparent viscous gel was obtained. However, in the case of LMO-W, the compound with water, the esterification reaction did not occur, owing to which the reaction temperature was maintained at approximately 60 °C. The resulting sol-gel was dried at 120 °C in an oven for 12 h, then ground. The calcination process was then continued at a temperature of 400 °C for 5 h, and at 800 °C for 10 h. The product was naturally cooled and ground, producing a final spinel LMO active material. There are several factors that can affect the sol-gel synthesis of high-quality products, such as the solvent type, concentration of the solution, precursor selection, pH, drying and calcination temperatures, catalysts, agitation, contents of a chelating agent, and time; other factors depend on the recipe for synthesis. Therefore, it is important to note that while the sol-gel synthesis method is a useful way to synthesize pure spinel LMO, the final product can be affected by many factors, and appropriate optimization of the synthesis conditions is therefore necessary. Sol-gel formation is highly sensitive to the reaction conditions, and it is necessary to develop a recipe that is convenient for our purposes.

### 4.3. Characterization of Materials

The morphologies and compositions of the powders were investigated using field emission scanning electron microscopy (FE-SEM; JSM-6701F, JEOL) and energy dispersive spectrometry (EDS) using an Ultim@MAX (OXFORD Instrument, UK) operated at 20 kV and samples were coated with a carbon thin film. X-ray diffraction (XRD; Miniflex, Rigaku; Cu Kα 1.5406 nm as radiation source) was used to characterize the phase structures with a scanning range 2θ of 10–90° and a scanning rate of 2° per min. X-ray photoelectron spectroscopy (XPS) measurements were performed with a Kratos Axis Ultra spectrometer to determine the balance states of Mn ions. The spectra of −5–1195 eV in the regions were measured with the pass energy of 187.35 eV and the step-width of 1.6 eV. The enlarged spectra in the Li1s, Mn2p, Mn3p, O1s, and C1s regions were measured with the pass energy of 20 eV and the step width of 0.125 eV. Fourier transform infrared (FT-IR—Nicolet, IS50, Thermo Fisher, Waltham, Massachusetts USA) spectra were used to determine the bonding nature of the sol-gels. The gels of the samples were collected before calcination at 450 °C and analyzed. The electrochemical performance of the powders was tested using two-electrode coin cells (CR 2032) with Li metal foil as the counter and reference electrodes. The working electrode was prepared by mixing 80 wt. % active material, 10% conductive carbon black (Imerys’s Ensaco 350P, Yeoksam-dong, Gangnam-gu, Seoul), and 10% binder (polyvinylidene fluoride, PVdF) in N-methyl pyrrolidone (NMP) solvent, and the obtained slurry was coated onto the AI foil current collector by casting. The coated slurry was dried at 110 °C for 12 h, and the electrode disks were punched and weighed. The active mass of the electrode was ~0.36 mg/cm^2^. The assembly process was performed in an Ar-filled glove box. A microporous polypropylene (PP) membrane was used as the separator, and a 1 M solution of LiPF_6_ in EC:DEC (1:1, vol. %) was used as the electrolyte. Galvanostatic charge–discharge tests (WDCS3000s, Won A-Tech, Yeogsam2-Dong, Kangnam-Ku Seoul Republic of Korea) were carried out at 0.5 C (theoretical capacity of 1 C is 148 mAh g^−1^) in a voltage range of 3.0 V to 4.35 V to investigate the electrochemical properties of the electrodes. Electrochemical impedance spectroscopy (EIS) was used to analyze the interfacial properties; EIS scans were conducted between 1 MHz and 10 MHz with a cut-off of 3.0 V after 100 cycles of the Galvanostatic charge–discharge tests. 

## 5. Conclusions

Cubic spinel LMO with an Fd-3m space group was successfully synthesized from two types of sol-gels (modified and unmodified metal complexes)—chelate and organic polymeric gels—without any contamination, and their synthesis mechanism, physical parameters, and electrochemical properties were investigated. This study highlights that synthesizing spinel LMO using a polymer-like gel structure made of ethylene glycol, as one part of the polymerization monomer, has several benefits including improved homogeneity. That is, the polymer-like gel structure can provide uniform distribution of Li, Mn, and O precursors, thereby leading to better homogeneity in the final product. Furthermore, modifying the polymerization process can help control the size of the particles formed during synthesis, thereby leading to more consistent particle sizes and better control over the final product. The polymer-like gel structure can provide a larger surface area for the reaction to occur, thereby increasing the overall reaction rate and improving the yield of the final product. Electrochemical results show that the LMO-G exhibits higher capacities for the initial cycles, approaching the expected high energy density due to the improved particle size and improvement of the homogeneity and uniformity of powder. Compared to LMO-E and LMO-G, LMO-W has a more stable cycle life, suggesting that LMO can provide an advantage in market demand without additional energy density improvements. However, for further developments of LMO, such as increasing the energy density, stable cycle life, and voltage, it is important to obtain pure phase and initially higher capacity LMO nanoparticles with uniform size distribution and high surface area; in this context, the proposed strategy of LMO-G is optimal. 

## Figures and Tables

**Table 1:** Physical parameters for LiMn_2_O_4_ materials synthesized using the citric acid-assisted method.

Powders	Particle Size, (D) (nm)	Lattice Constant, (a) (Å)	The Volume of the Unit Cell (V) (Å)^3^	Peak Intensity Ratio (I_111_/I_311_) (I_311_/I_400_)	Peak Intensity Ratio (I_400_/I_111_) (I_440_/I_111_)	Strain, (e) (×10^−6^)
LMO-W	351	8.23	557.5	1.88 1.09	0.50 0.25	3.57
LMO-E	312	8.22	555.5	1.81 1.02	0.52 0.27	2.89
LMO-G	219	8.21	553.3	1.81 1.05	0.53 0.26	2.62

## Data Availability

Not applicable.

## Sample Availability

Samples of the compounds are available from the authors.

## Appendix A

**Table A1:** LiMn_2_O_4_ powder synthesized by a citric acid chelate agent of sol-gel method.

No. Precursors	Synthesis Conditions			Particles Characteristics		Electrochemical Properties					Ref
	Gel Structure	Calcination Parameters		Morphology	Particle Size	C-Rate	Voltage Range	Initial Discharge Q	Capacity Retention	Testing Temperature	
		Temperature (°C)	Duration (h)		(nm)	(C)	(V)	(mAh g^−1^)	(mAhg^−1^/n^−1^)	(°C)	
LiAC MnAC	W	800	10	octahedral	419	0.5	3.0–4.35	121.3	82/100	RT	our
LiAC MnAC	ET	800	10	octahedral	305	0.5	3.0–4.35	126.6	79/100	RT	our
LiAC MnAC	EG	800	10	octahedral	253	0.5	3.0–4.35	141.6	70/100	RT	our
LiAC MnAC	W	750	10	agglomerate	200–600	1	3.5–4.30	117.5	93.4/100	25	[76]
LiAC MnAC	W	750	12	-	-	0.1	-	57.57	30.92/60	-	[77]
LiAC MnAC	W	850	15	spherical	150	0.5	3.0–4.50	135.0	81.0/100	30	[60]
LiAC MnAC	W	750	12	octahedral	200	1	3.4–4.50	127.0	115/100	RT	[78]
LiAC MnAC	W	850	15	-	-	1	3.0–4.50	125.0	69.0/100	RT	[79]
LiAC MnAC	W	800	12	cubic facets	400	0.2	3.5–4.30	121	55.00/80	RT	[80]
LiAC MnAC	W	800	16	-	-	0.2	3.0–4.50	126.6	98.75/70 (1C)	RT	[81]
LiAC MnAC	W	850	7	polyhedral	100–300	1	3.0–4.30	110	72.00/50	RT	[82]
LiAC MnAC	W	800	10	grains	-	1	0.3–1.05	103	-	RT	[83]
LiAC MnAC	W	799	10	irregular	100–500	1	3.0–4.50	83.7	75.0/100	RT	[84]
LiAC MnAC	W	800	12	-	300–400	0.5	3.0–4.50	-	85/35	25	[10]
LiAC MnAC	W	300–800	-	-	<100	-	-	-	-	-	[85]
LiAC MnAC	W	600	5	-	500–1000	0.1	3.0–4.30	132	125/50	RT	[86]
LiNO_3_ Mn(NO_3_)_2_	W	750	5	grains	50	0.2	4.30	120	87/300 (1C)	-	[56]
LiNO_3_ Mn(NO_3_)_2_	W	800	10	-	-	-	3.6–4.30	127.4	105.63/6	RT	[13]
LiOH MnAC	W	780	12	-	-	0.5	3.1–4.40	-	93.7/100	RT	[87]
LiAC MnAC	ET	300	24	Some agglomerate	30–50	1	3.0–4.30	111.4	88.1/100	25	[88]
LiAC MnAC	EA	850	14	-	520–1240	0.5	3.5–4.20	47.00	33.00/40	25	[89]
LiOH MnAC	ET	750	18	Serious agglomerate	200	0.5	3.2–4.35	134.0	108.5/30	RT	[61]
LiAC MnAC	ET	300	24	-	30–50	1	3.0–4.50	110	90.3/100	RT	[90]
LiNO_3_ Mn(NO_3_)_2_	ET	700	12	irregular	100	0.5	3.3–4.50	114	-	RT	[91]
LiAC MnAC	ET	800	24	-	-	-	3.5–4.20	105	84/15	RT	[11]
LiAC MnAC	EG	450–700	-	grains	100–200	-	-	-	-	-	[92]
LiNO_3_ Mn(NO_3_)_2_	EG	800	22	-	10–100	-	3.0–4.20	130	98/50	RT	[93]

C—148 (mAhg^−1^); RT—room temperature; ET—ethanol; W—water; EG—ethylene glycol; EA—2-ethylhexanoic acid.
